# Molecular Dynamics Simulation and Essential Dynamics of Deleterious Proline 12 Alanine Single-Nucleotide Polymorphism in PPAR*γ*2 Associated with Type 2 Diabetes, Cardiovascular Disease, and Nonalcoholic Fatty Liver Disease

**DOI:** 10.1155/2022/3833668

**Published:** 2022-05-02

**Authors:** Somayye Taghvaei, Leila Saremi

**Affiliations:** ^1^Department of Medical Biotechnology, National Institute of Genetic Engineering and Biotechnology, Tehran, Iran; ^2^Department of Biology, Science and Research Branch, Islamic Azad University, Tehran, Iran

## Abstract

*Background*. Peroxisome proliferator-activated receptor-*γ* (*PPARγ*) gene is located at 3p25 position. PPAR*γ* functions as the master regulator of glucose homeostasis and lipoprotein metabolism, and recent studies have reported that it is involved in various metabolic diseases such as diabetes mellitus, hyperlipidemia, coronary artery disease (CAD), and nonalcoholic fatty liver disease (NAFLD). PPAR*γ*1 and PPAR*γ*2 are necessary for the development of adipose tissue and insulin sensitivity regulation. But PPAR*γ*2 is the isoform that was controlled in response to nutrient intake and obesity. *Methodology*. In this study, we used computational techniques to show the impact of Pro12Ala polymorphism on PPAR*γ*2. The 3-D structure of PPAR*γ*2 was modeled using I-TASSER server. The modeled structure was validated with the ZLab server, and the mutation was created with SPDB viewer. Stability prediction tools were used. Molecular dynamics simulation (MDS) was used to understand the structural and functional behavior of the wild type and mutant. Essential dynamics was also applied. *Results and Conclusions*. Stability prediction tools were showed that this mutation has a destabilizing effect on the PPAR*γ*2 structure. The RMSD, RMSF, Rg, SASA, and DSSP were in line with H-bond results that showed less flexibility in the mutant structure. Essential dynamics was used to verify MDS results. Pro12Ala polymorphism leads to rigidity of the PPAR*γ*2 protein and might disturb the conformational changes and interactions of PPAR*γ*2 and results in type 2 diabetes mellitus (T2DM), CAD, and NAFLD. This study can help scientists to develop a drug therapy against these diseases.

## 1. Introduction

Peroxisome proliferator-activated receptor-*γ* (PPAR*γ*) has a key role in adipogenesis, liver and muscle responses to glucose, and pancreatic b-cell function [1]. PPAR*γ* regulates glucose and lipid metabolism. PPAR*γ* has an immune and inflammation suppressive function, which results in an antiatherogenic effect [2, 3]; thus, genetic variation in the PPAR*γ* may regulate individual susceptibility to type 2 diabetes mellitus (T2DM) and coronary heart disease [4]. Alternative splicing of PPAR*γ* results in four isoforms PPAR*γ*1, PPAR*γ*2, PPAR*γ*3, and PPAR*γ*4 in which PPAR*γ*2 is primarily expressed in the adipose tissue [5]. PPAR*γ*2 is a transcription factor that is formed by alternative splicing. Single-nucleotide polymorphisms (SNPs) are widely divided into two distinct clusters, synonymous (sSNPs) and nonsynonymous SNPs (nsSNPs). The nonsynonymous SNPs are further divided into missense mutations and nonsense mutations. The coding synonymous SNPs have a low effect on the protein structure, while the nonsynonymous SNPs have a great impact on the protein structure and higher risk of diseases [6]. The most common single-nucleotide polymorphism PPAR*γ*2 rs1801282 (C>G Pro12Ala) was identified by Yen et al. in 1997 that reduces transcription of PPAR*γ*2 [7, 8]. The Pro12Ala polymorphism in PPAR*γ*2, a Pro-to-Ala substitution at codon 12, leads to a reduction in both DNA binding and transcriptional activity *in vitro*. Ala12 allele carriers have a significant improvement in insulin sensitivity [2]. Frequency of this mutation in the gnomAD database is 0.6 with 1759 being homozygous and 27634 being heterozygous.

Increasing experimental studies have investigated the relationship between Pro12Ala polymorphism of *PPARγ2* gene and various diseases such as T2DM, insulin sensitivity, obesity, cardiovascular diseases, Alzheimer's disease, and cancer [3]. Nonalcoholic fatty liver disease (NAFLD) is a progressive liver disease that is determined by dyslipidemia, obesity, hypertension, hypercholesterolemia, type 2 diabetes mellitus, cirrhosis, liver failure, insulin resistance, and hepatocellular carcinoma [9]. Several researchers reported that Pro12Ala polymorphism was significantly associated with NAFLD [10–13] in different populations.

Coronary artery disease (CAD) is the most common cause of death among diabetic patients. Different studies have also shown which PPAR*γ*2 Pro12Ala polymorphism was associated with CAD [8, 14]. Saremi et al. found PPAR*γ*2 (c.34G>C, Pro12Ala) is considerably associated with higher risk NAFLD [9] in T2DM patients in Iranian population. These findings suggest a high possibility of involvement of Pro12Ala polymorphism in the risk for CAD, NAFLD, and T2DM.

Computational methods were being applied to the study of the structural and functional effect of point mutation at the molecular level. In this investigation, we also implemented multiple computational methods to identify the effect of rs1801282 (C>G Pro12Ala) mutation on PPAR*γ*2 protein. The rs1801282 (C>G Pro12Ala) SNP in the PPAR*γ*2 coding region has an impact on the related disease phenotype, and stability tools were used to the survey of this polymorphism. The studies have shown that in silico methods can be applied to survey protein structure and function [15–19]. Molecular dynamics simulation (MDS) is an important tool for understanding the effect of mutations on the protein structure, as it provides information about the protein at the atomic level on a reasonable time scale. In order to check (i) whether mutant (12Ala PPAR*γ*2) has an impact on the conformation of the PPAR*γ*2, (ii) whether the mutant structure deviates from the native PPAR*γ*2, and (iii) whether mutant changes flexibility of PPAR*γ*2. We have carried out molecular dynamics simulation of MT (mutant type) and WT (wild type) to predict pathogenic phenotype associated C>G Pro12Ala SNP and further to reveal the conformational flexibility of the mutant PPAR*γ*2 through extensive MDS. At the end, essential dynamics (ED) was applied to the study of the mutant and wild-type proteins. In general, our results provide strong evidence of main conformational drift occurring in Pro12Ala polymorphism as compared to the wild type.

## 2. Materials and Methods

### 2.1. Modelling of Protein

rs1801282 (C>G Pro12Ala) SNP was retrieved from dbSNP database (http://www.ncbi.nlm.nih.gov/projects/SNP/, access date: February 19, 2020) [20] for our computational analysis. The amino acid sequence of PPAR*γ*2 with UniProt ID: P37231 was downloaded from UniProt for the study.

We explored RCSB PDB, but there was not a crystallographic structure that included this polymorphism site. Therefore, human PPAR*γ*2 protein (with 505aa) was modeled by an automated protein structure prediction program (I-TASSER) [21]. The modeled structure of PPAR*γ*2 protein was evaluated by the ZLab server (https://zlab.umassmed.edu/bu/rama/index.pl).

We replaced the wild-type protein residue with itself (used as wild-type) and with Alanine (used as mutant) using SPDB viewer [22]. Then, wild type and mutant are minimized with YASARA. In the next step, the effect of this polymorphism on PPAR*γ*2 stability was explored by SNP tools.

### 2.2. Stability Prediction

Since a missense polymorphism causes alteration of the protein structure and function, therefore, we predicted the protein stability. A number of recent studies have verified implementing multiple bioinformatics tools and algorithms which increase the accuracy of the results [23–29]. To evaluate the effect of the amino acid substitution at position 12 on the stability of wild-type PPAR*γ*2, we used the following stability predictor tools. MUpro is an assembly of programs with machine learning that computes the protein stability and changes based on sequence data, especially when the tertiary structure is not subjected. This approach dominates significant restrictions on previous approaches based on the tertiary structure [30]. DynaMut can perform rapid analysis of the protein stability and dynamics coming from alterations in vibrational entropy [31]. DUET also predicts the effect of point mutations on the protein stability through an embedded computational approach [32]. The mCSM calculates the consequences of missense polymorphisms on the stability of protein, protein-protein binding, and protein-DNA interaction [33]. I-Mutant 2.0 calculations are based on the protein structure or the protein sequence or are based on the prediction of the protein stability of missense variants [34]. The SNAP server was also used. To investigate the mechanism of structural consequences of Pro12Ala mutation on PPAR*γ*2, we performed molecular dynamics simulation.

### 2.3. Molecular Dynamics Simulation

#### 2.3.1. MD Simulation

To evaluate the deleterious effect of Pro12Ala mutation on the interaction of PPAR*γ*2 protein, we performed molecular dynamics simulation using the actual tool of GROMACS [35]. MDS was carried out with the parallel version of PME in the GROMACS program. The 10 Å nonbonded cut-off was considered. MDS was started with solvation within a dodecahedron-shaped water cage and the 1 nm distance between the cage edges and protein periphery. System neutralization was done with an addition 13 NA ions. Then, 1000 steps of energy minimization were done. Molecular dynamics simulation was performed at 300 k, 1 atm pressure, and GROMOS53a6 force field using GROMACS 4.6.5 (http://www.gromacs.org/). Before MDS run, the structure was gained to 300 K the temperature and equilibrated during 100 ps under conditions of constant volume and temperature (NVT). Next, the system was switched to constant pressure and temperature (NPT) and was equilibrated for 100 ps. 50 ns MDS of WT and MT PPAR*γ*2 in 25 × 10^6^ steps individually were applied. The cutoff radius of protein-solvent intramolecular hydrogen bonds was 0.35 nm. The periodic boundary condition function was carried out by the leap-frog algorithm with a 2 fs time step and every 500-step structural snapshots (1 ps) [36].

#### 2.3.2. Analysis of Molecular Dynamics Trajectories

Structural deviation analyses of the mutant and wild-type proteins such as root-mean-square deviation (RMSD), root-mean-square fluctuation (RMSF), solvent accessible surface area (SASA), gyration radius (Rg), hydrogen bonds (H-bond), and the secondary structure of the protein (DSSP) were computed using g_rmsd, g_rmsf, g_sasa, g_hbond, g_gyrate, and do_dssp built-in functions of GROMACS package. GRACE software was used for plotting of graphs (http://plasma-gate.weizmann.ac.il/Grace/).

### 2.4. Essential Dynamics

Essential dynamics, known as Principal Component Analysis (PCA), can show the collective atomic motion of the mutant and wild-type proteins by the GROMACS tool. Principal component analysis was computed using g_covar and g_anaeig built-in functions of the GROMACS package. PCA is a standard protocol for the characterization of eigenvectors and the projection across the first PC1 and PC2 [6].

## 3. Results

### 3.1. Modeling SNP Location on Protein Structure

**Figure 1:**
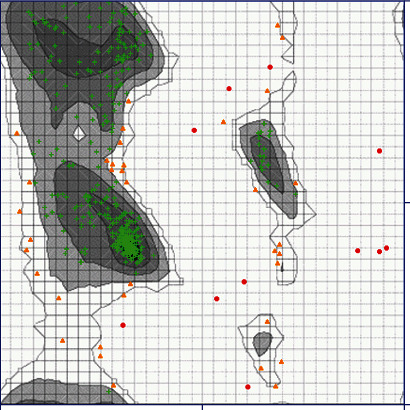
The Ramachandran plot of evaluation 3D structure was modeled using ZLab server; green: highly preferred conformations, delta ≥ −2; brown: preferred conformations, −2 > delta ≥ −4; and red: questionable conformations, delta < −4.

The modeling with I-TASSER gave five models. The best structure with high confidence score was collected and used for further investigations (*C*‐score = −2.38 and estimated TM-score = 0.44 ± 0.14). Model 1 with the highest C-score was selected for further studies. Model 1 was validated by the ZLab server that showed 97.6% of the amino acids of modeled structure in the allowed area (Figure 1), meaning that this model is suitable for further study. The amino acid replacement was also done using SPDB viewer. In the next step, the effect of Pro12Ala polymorphism on the structure and function of PPAR*γ*2 was exhibited by stability prediction tools.

### 3.2. Stability Prediction

Most of disease-associated polymorphisms have a significant influence on protein stability. To characterize the impact of Pro12Ala SNP on the PPAR*γ*2 structure and function, several computational prediction tools were used. IMutant2.0 has predicted Pro12Ala polymorphism decreases the stability of PPAR*γ*2. mCSM, DUET, and DynaMut also showed the destabilizing impact of Pro12Ala polymorphism on PPAR*γ*2 with DDG (-1.112, -0.6145, and -0.677 cal/mol, respectively). SNAP was also shown, in which this polymorphism is pathogenic with 85% expected accuracy. In the next step, we studied molecular dynamics simulation of the mutant and wild type.

### 3.3. MD Simulation

**Figure 2:**
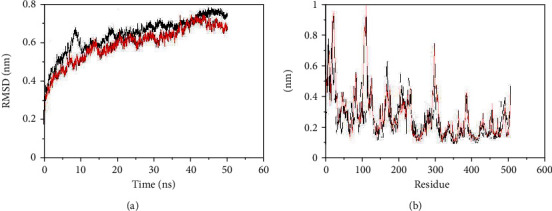
GROMACS analysis of Backbone RMSD and RMSF as a function of time for the mutant and wild-type at 50 ns molecular dynamics simulation; red: mutant; black: wild type; A: RMSD; B: RMSF.

Now, computational analysis is a roadmap to define a standard disease-specific SNP at the molecular level. In this study, we examined rs1801282 (C>G Pro12Ala) PPAR*γ*2 which is related to several diseases especially CAD, NAFLD, and T2DM. MDS approaches are also extensively used to report the structural consequences of the deleterious predicted point mutations. We calculated respective C*α*-root mean square deviation (C*α*-RMSD) for simulations that are plotted in Figure 2(a). WT RMSD plot equilibrated in 0.74 nm while MT RMSD plot equilibrated in 0.65. It represents mutation result in less flexibility of PPAR*γ*2. To understand how mutant affects the dynamic behavior of the residues and to examine the cause of conformational drifts observed in RMSD, C*α*-root mean square fluctuation (C*α*-RMSF) of WT and MT amino acid residues were calculated and are plotted in Figure 2(b). Lower fluctuations were seen in MT compared to WT. Then, this mutation decreases the flexibility of the PPAR*γ*2 protein.

**Figure 3:**
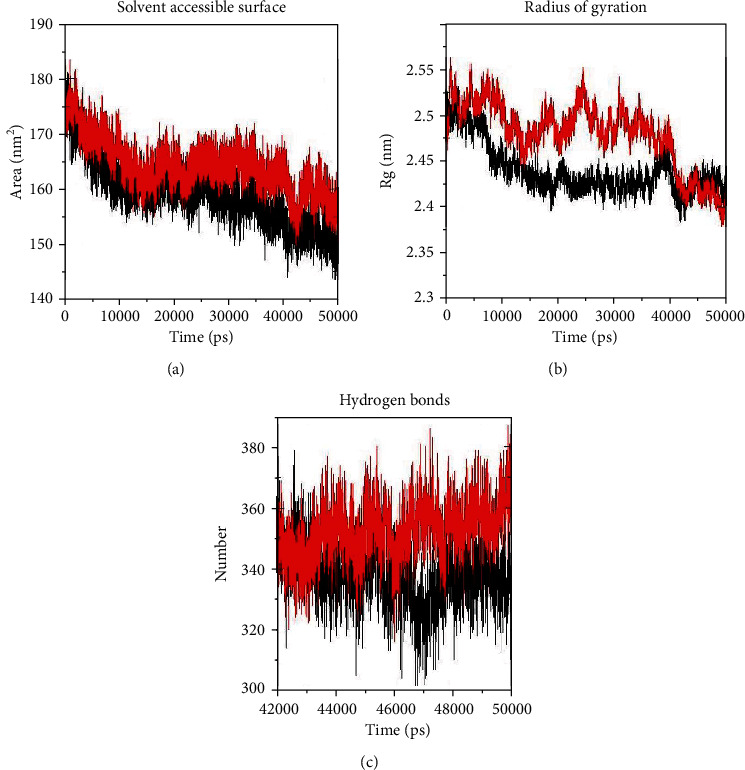
GROMACS analysis of Rg, SASA, and intramolecular hydrogen bonds of C*α* atoms for the mutant and wild type in the PPAR*γ*2 protein at 300 K. (a) Rg, (b) SASA, and (c) intramolecular hydrogen bonds. Mutant was shown in black and wild type in red.

SASA is a representative of the grade in which an amino acid interacts with its environment (solvent and protein) [37]. An increase or decrease in SASA plot displays changes in the subjected amino acid residues, so affecting the protein tertiary structure. Results of the analysis showed that SASA of WT was 152 nm^2^ and SASA of MT was 159 nm^2^, which showed a less average total SASA in WT compared to the MT (Figure 3(a)). Rg is also a parameter to explain the equilibrium conformation of a total system specifically in analyzing proteins. It is indicative of the compression level of the protein structure; i.e., polypeptide chain was folded or was unfolded [36]. We observed a more decrease in Rg of MT compared to WT (Figure 3(b)). WT Rg decreased to 2.41 nm while MT Rg decreased to 2.38 nm; then, MT has more compactness and more rigidity structure.

One of the main factors that account for maintaining the stable conformation of a protein is hydrogen bonding [36]. We have performed the NH bond analysis of WT and MT during simulations that are plotted in Figure 3(c). Results showed a significant difference in intramolecular hydrogen bond pattern between WT and MT. A greater average number of hydrogen bonds was observed in the MT (376) compared to the WT (340) during simulation, indicating that the occurrence of this mutation may lead to a more compact conformation and rigidity of PPAR*γ*2 protein (Figure 3(c)).

**Figure 4:**
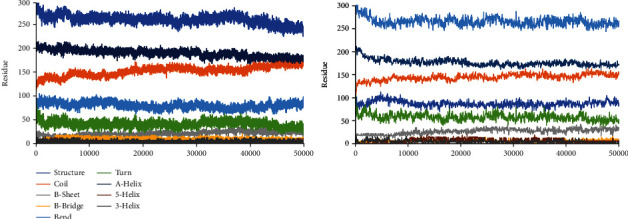
Secondary structural elements for the mutant and wild type in the PPAR*γ*2 protein. The color of each secondary structure is displayed in the legend.

In the end, the secondary structure of the mutant and wild-type proteins was considered that indicated negligible differences (Figure 4). Generally, this mutation decreases flexibility and stability of PPAR*γ*2 which results in the decrease of PPAR*γ*2 expression.

### 3.4. Essential Dynamics Analysis

**Figure 5:**
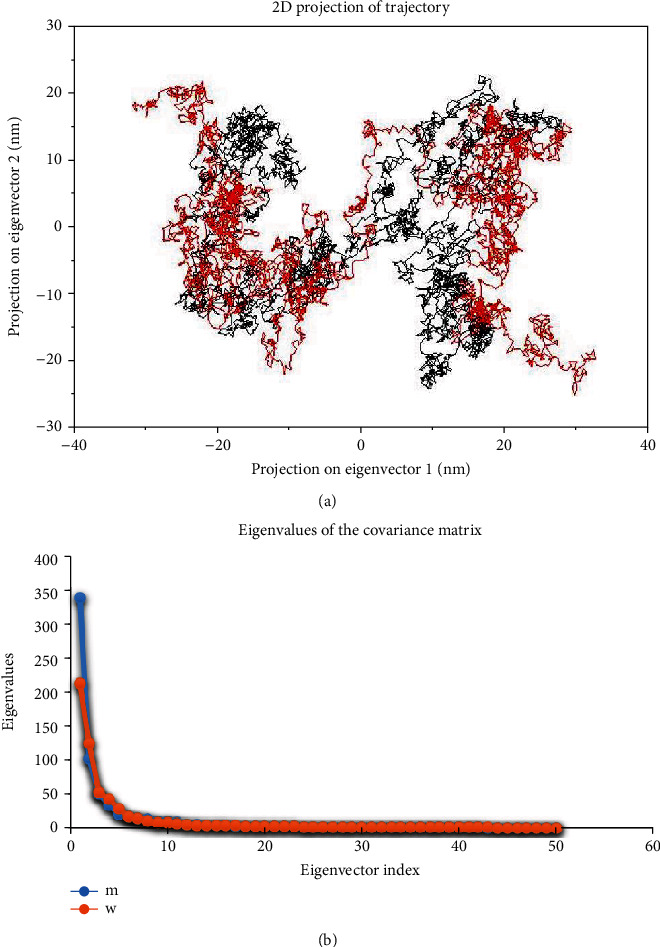
Principal component analysis. (a) Projection of the motion for wild-type (black) and mutant (red) in phase space along the PC1 and PC2; (b) eigenvalues vs. eigenvector index plot were plotted for the first 50 eigenvector, wild type (red), and mutant (blue).

In this step, we used essential dynamics analysis to obtain the dynamics of the mutant and wild-type proteins. The projection of trajectories of the mutant and wild-type proteins during the essential dynamics in the phase space along the first two principal components (PC1, PC2) at 300 K is plotted in Figure 5. It predicts the large-scale collective motions for the mutant and wild-type of the PPAR*γ*2 protein. PCA analysis showed in which, due to mutation, the structural dynamics is changing.  Figure 5(a) plot clearly indicates the mutant occupied less space in phase space while the wild-type occupied more space. The first 50 eigenvectors were selected to compute concerted motions (Figure 5(b)). The eigenvalues were obtained from the diagonalization of the covariance matrix of atomic fluctuations. We observed increased flexibility of the wild type than the mutant; then, Pro12Ala polymorphism causes the rigidity. The PCA analysis results agree with the results from MDS.

## 4. Discussion

**Figure 6:**
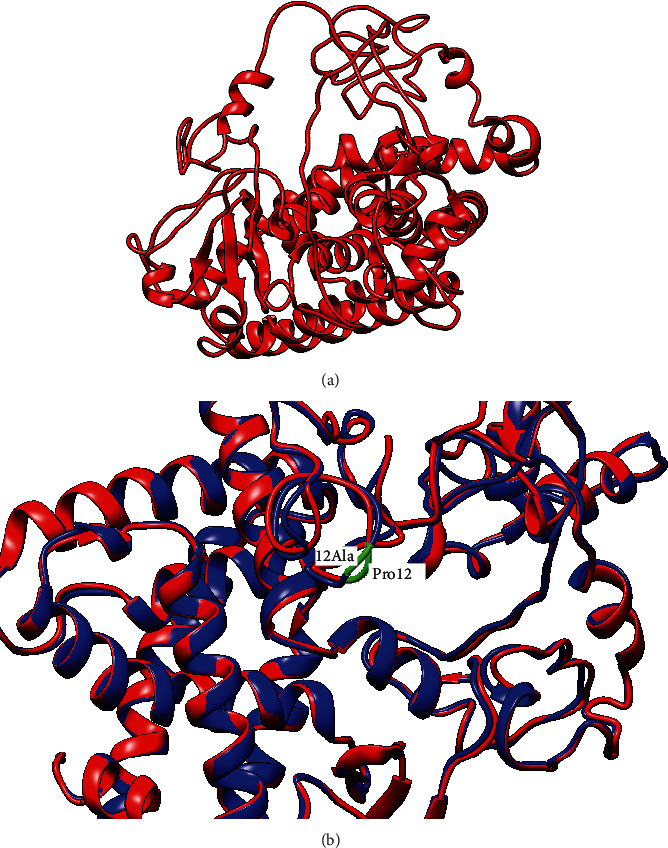
Superimposition of secondary structures of mutant and wild-type by UCSF Chimera: (a) whole PPAR*γ*2 protein; (b) mutation site, Pro12 allele (red), and 12Ala allele (blue). Mutation site was highlighted in green.

Studying mutations can help to comprehend the disease's molecular mechanism which is associated with their inheritance modes and to exhibit how genetic mutations can show various clinical properties through interference in different protein interactions [38]. The polymorphism rs1801282 (c.34C>G) in codon 12 of the *PPARγ2* gene, which results in the substitution of Proline with Alanine (Pro12Ala), was found to be related to higher insulin sensitivity and diminished risk of T2DM and diabetic nephropathy [39–42] as well as CAD and NAFLD. PPAR*γ*2 regulates the transcription and the expression of several target genes, which have been shown to be implicated in adipocyte differentiation, lipid and glucose metabolism, and atherosclerosis [9]. Therefore, PPAR*γ*2 is the main candidate gene for obesity, T2DM, CAD, and NAFLD diseases. Adult diabetic patients have an increased risk of mortality due to heart disease compared with those without diabetes [21]. Dyslipidemia, obesity, and hypertension lead to enhancing CAD risk in T2DM patients [1, 22]. We superimposed wild-type and mutant proteins and showed mutation site in Figure 6.

We used a computational approach for the survey of (Pro12Ala) polymorphism. Initially, PPAR*γ*2 was modeled, mutated, and then energy minimized. Then, in silico prediction tools including IMutant2.0, Mupro, DUET, DynaMut, and mCSM were indicated in which the Pro12Ala mutation affects the protein structure and function. Kumar et al. showed both p.S380N and p.R423H mutations destabilize ALDH3A2 [38], and Kumar et al. also indicated W148R, F161C, and L171R mutations in FLN (filamin) B result in the loss of stability and consequently leading to AOI, LS, and BD phenotypes [27]. Exact MDS enables scientists to observe many important biochemical phenomena that cannot be observed when the folding of the proteins into their natural three-dimensional structure [43]. This technique is used to understand the dynamical behavior of the proteins at different time scales from rapid internal motions to slow conformational changes or even protein folding processes [44, 45]. We have performed MDS to study the structural and dynamic effects of this mutation in comparison to the wild-type protein. Simulation results revealed a detailed consequence of the Pro12Ala mutation on the PPAR*γ*2 protein that may provide insight for therapeutic approaches, especially in T2DM and CAD or NAFLD.

The present study will offer an in-depth insight into the genotype-phenotype association of deleterious SNP rs1801282 (C>G Pro12Ala) in PPAR*γ*2. The flexibility loss is specifically observed in RMSD, RMSF, and Rg plots that showed Ala12 allele has a major impact on the structural conformation of the PPAR*γ*2 protein. A higher number of H-bonds were observed in the PPAR*γ*2 mutant than in the wild-type protein which might lead to a rigid structure of PPAR*γ*2. Previously, we indicated which G482S leads to rigidity and instability of PPARGC1A protein [6]. Kamaraj and Purohit also showed R326H and R356Q resulting in rigidity of tyrosinase-related protein-1 (TYRP1) protein which might disturb the structural conformation and catalytic function of the structure and also play a significant role in inducing Oculocutaneous albinism type III (OCA3) [46]. In the study of Kumar et al. Y63H mutation was also shown with more hydrogen bonds disrupt the wild-type conformation of ATP binding region in CENP-E motor domain [47]. Kumar et al. also suggested that W148R, F161C, and L171R mutations in FLNB might cause the structure to be rigid due to more hydrogen bonds [27].

ED analysis was used for more surveys and was showed a loss of flexibility caused to this polymorphism. Overall, the present computational approach will provide a comprehensive view of the pathogenic mechanism of rs1801282 SNP, and it may serve as a useful model for predicting the effect of SNPs in other proteins. The results reported in this study elucidate the role of Pro12Ala in PPAR*γ*2 which may provide a useful information for the design of the PPAR*γ*2 mutation-based therapeutic strategies against T2DM.

## 5. Conclusion

Dysregulation of metabolism is involved in obesity and other diseases like type 2 diabetes mellitus and cardiovascular diseases, which are associated with abnormalities of PPAR*γ*2. *PPARγ*2 overexpression has been reported to improve type 2 diabetes metabolic and other related conditions [48]. As we mentioned, several researches on PPAR*γ*2 Pro12Ala polymorphism have indicated this polymorphism is pathogenic in different diseases including T2DM, insulin sensitivity, obesity, cardiovascular diseases, Alzheimer's disease, and cancer [3]. In this study, we also provided evidence of deleterious conformational changes in the PPAR*γ*2 protein that has a significant role in creating disease-associated phenotypes. Ala12 allele represented which leads to disease by changing the structural conformation of PPAR*γ*2.

The stability of a protein is required for its correct function [49–53]. We exhibited a destabilizing effect of this polymorphism using the stability prediction tools. Molecular dynamics simulation was indicated the difference in the dynamics of the PPAR*γ*2 mutant and wild-type proteins. The dynamics of the protein are dependent on the structural flexibility of PPAR*γ*2, and H-bonds are essential to stabilize the protein structure [6]. MD results displayed the decreased flexibility of the Pro12Ala polymorphism structure. The ED analysis also showed this mutation changes the original structural geometry and the structural conformation of PPAR*γ*2 protein, resulting in the loss of the protein function. This suggests that, due to mutation, the structure became more rigid and that this might result in instability and decreasing PPAR*γ*2 expression in patients with related diseases.

The results obtained from this study would facilitate wet-lab researches to develop a potent drug therapy against PPAR*γ*2. The results of this study report the role of Pro12Ala polymorphism in PPAR*γ*2 and may provide useful information for the design of Pro12Ala polymorphism-based therapeutic strategies against especially CAD, NAFLD, T2DM, insulin sensitivity, obesity, cardiovascular disease, Alzheimer's disease, and cancer.

## Data Availability

Data is available on request.
